# Health coping strategies of the people vulnerable to climate change in a resource-poor rural setting in Bangladesh

**DOI:** 10.1186/1471-2458-13-565

**Published:** 2013-06-10

**Authors:** Md Aminul Haque, Aji Budi, Ahmad Azam Malik, Shelby Suzanne Yamamoto, Valérie R Louis, Rainer Sauerborn

**Affiliations:** 1 Institute of Public Health, Heidelberg University, 69120, Heidelberg, Im Neuenheimer Feld 324, Germany; 2 Department of Population Sciences, University of Dhaka, 1000, Dhaka, Bangladesh; 3 University Institute of Public Health (UIPH), The University of Lahore (UOL), Lahore, Pakistan

**Keywords:** Health coping strategies, Choice of care, Unqualified providers, Health expenditure, Health insurance, Climate sensitive diseases, Resource poor setting in Bangladesh

## Abstract

**Background:**

Among the many challenges faced by the people of Bangladesh, the effects of climate change are discernibly threatening, impacting on human settlement, agricultural production, economic development, and human health. Bangladesh is a low-income country with limited resources; its vulnerability to climate change has influenced individuals to seek out health coping strategies. The objectives of the study were to explore the different strategies/measures people employ to cope with climate sensitive diseases and sickness.

**Methods:**

A cross-sectional study was conducted among 450 households from Rajshahi and Khulna districts of Bangladesh selected through multi-stage sampling techniques, using a semi-structured questionnaire supplemented by 12 focus group discussions and 15 key informant interviews.

**Results:**

Respondents applied 22 types of primary health coping strategies to prevent climate related diseases and sickness. To cope with health problems, 80.8% used personal treatment experiences and 99.3% sought any treatments available at village level. The percentage of respondents that visited unqualified health providers to cope with climate induced health problems was quite high, namely 92.7% visited village doctors, 75.9% drug stores, and 67.3% self-medicated. Ninety per cent of the respondents took treatment from unqualified providers as their first choice. Public health facilities were the first choice of treatment for only 11.0% of respondents. On average, every household spent Bangladesh Currency Taka 9,323 per year for the treatment of climate sensitive diseases and sickness. Only 46% of health expenditure was managed from their savings. The rest, 54% expenditure, was supported by using 24 different sources, such as social capital and the selling of family assets. The rate of out-of-pocket payment was almost 100%.

**Conclusion:**

People are concerned about climate induced diseases and sickness and sought preventive as well as curative measures to cope with health problems. The most common and widely used climate health coping strategies among the respondents included self-medicating and seeking the health service of unqualified private health care providers. Per family spending to cope with such health problems is expensive and completely based on out of pocket payment. There is no fund pooling, community funding or health insurance program in rural areas to support the health coping of the people. Policies are needed to reduce out-of-pocket payment, to improve the quality of the unqualified providers and to extend public health services at rural areas and support climate related health coping. Collection of such knowledge on climate related health coping strategies can allow researchers to study any specific issue on health coping, and policy makers to initiate effective climate related health coping strategies for climate vulnerable people.

## Background

Bangladesh’s vulnerability to climate change has been recognized in global media accounts: it has been referenced in United Nations’ reports [1,2], has made headlines in national [3] and international [4,5] reports, and has been the center of concern in many journal articles [6-8]. The impending effects of climate change with potentially devastating consequences have drawn the highest attention at the ‘global, national and regional level during the decades’ [9]. Climate change and its negative effect on human beings is one of the greatest challenges for the global community. The Inter-governmental Panel on Climate Change (IPCC) repeatedly claimed that “climate change currently contributes to the global burden of disease and premature deaths”[10]. The world community has recognized that climate change affects human health negatively both directly and indirectly and can cause long-term effects [11]. It affects individuals, communities and societies as a whole [12].

Climate change has been identified as one of the major threats to human health of this century because of its potential effects on vector or water-borne diseases, cold spells, extreme heat, food and water scarcity and extreme climate variability and population displacement [13]. The majority of such health problems are especially unfavorable for vulnerable populations [14] and may increase global health disparities [15]. The World Health Organization (WHO) anticipates that climate change will cause abrupt and severe storms, floods and heat weaves in the upcoming years, and this will affect the most fundamental determinants of health [16,17]. Globally the frequency, severity and irregularities of natural disasters have tripled since the 1960s [17]. Although climate change poses a severe threat to human health, it has received relatively little attention among scientists and policy makers [13].

Although the impact of climate change on human health will be global, the health consequences will be distributed unequally across regions, occupation, gender, and age [17], and vary depending on community vulnerability level [9,18,19]. People from low and middle income countries are expected to be the most vulnerable to climate change and experience the greatest impact on health [20-22]. A WHO estimate projected globally an excess of 150,000 annual deaths due to changes in the world’s climate relative to the climate baseline of 1961–1990 [23,24].

In Bangladesh, where a large proportion of the population is vulnerable to climate change, health impacts are expected to take place through a variety of ways, including an increase of water and vector borne diseases and of health problems in general [6,7,25-27]. For example, southern Bangladesh is in a low-lying delta, making it vulnerable to sea level rise, severe storm-surges, floods and salinity intrusion. It is projected that a 1.5 meter rise in the sea level will inundate about 16% land of the southern part of Bangladesh, where about 17 million people live [28]. Almost every household of three districts of southern part of Bangladesh were severely affected by the cyclone “Aila’ in 2009 [28]. The average annual death toll in Bangladesh is about 8,241, due to extreme climatic events [29]. Projected extreme climatic events, such as droughts, cyclones, floods, tidal-surges, heat waves, cold spells, directly and indirectly affect major determinants of health and increase the occurrence of different diseases and sickness [28,30]. The Climate Change Cell (CCC) of Bangladesh noted that incidences of major climate sensitive diseases (i.e. diarrhea, skin diseases, malaria, mental disorders, dengue) have increased during last decade in Bangladesh [31]. A number of diseases like normal colds/coughs/fevers, dysentery, headaches, diarrhea, skin diseases, burning sensations, conjunctivitis, jaundice/hepatitis-B, skin burns/blistering, asthma, psychological disorders, typhoid, pox, weight loss, malnutrition related diseases, rheumatism/aching, pneumonia, measles, heatstroke, malaria, dengue etc., can be influenced by extreme climate events in Bangladesh [6,32]. As an immediate response to this increased health burden, people need to seek different steps and measures to improve the health situation. Policy makers also need to know the extent of health vulnerability and the strategies people use to avert increased sickness and diseases to formulate an effective program of action in the health sector for the climate vulnerable people of the country. Very little attention has been given by the research community in Bangladesh to investigate climate related health vulnerability and the diverse responses to cope with it. Efforts to study these human health risks remain very inadequate in Bangladesh [15]. Given the impending consequences of climate change for the people in Bangladesh, important areas of research are (i) increasing the understanding of community level health systems’ capacity to deliver health services, and (ii) individual capacity to cope with climate-related health problems. The objective of this study was to explore what people do to avert climate-induced health problems in resource-poor settings in Bangladesh. Specifically the study explored the various strategies people adopted to cope with increased climate-induced sickness and diseases.

## Methods

Health coping strategies of climate vulnerable people were assessed by using a mixed method research design as described in the existing literature [32-34]. A concurrent triangulation method was used for the study, in which qualitative and quantitative data were collected simultaneously. The findings were integrated into the results section. Data were collected from two villages, Dhuroil and Sachibunia, between September 2010 and March 2011. The villages were selected randomly. Dhuroil was located in the Rajshahi district in the northern part of Bangladesh, and the other, Sachibunia, in the Khulna district in the south. Based on the national statistics, overall socio-cultural, educational, occupational, and farming practices of the study areas were similar [31,35,36]. Both villages were well connected with the district headquarters. The administration of Bangladesh is divided into several hierarchal unites. These units include Division, District, Upazila and Union. Each village was serviced by a Union Family and Health Welfare Center (UFHWC) which was the first tier of government-owned health care system at the village level. This was the only public primary health care facility available to the villagers. The UFHWC was operated by one medical doctor, one paramedic and several nurses. It was open from 9am-5pm. The health center provided primary health care, antenatal care, checkup and consultations.

Two villages were selected randomly to obtain a wide-range of health coping strategies for climate-sensitive diseases among household members at community level of the country. Detail of the sample size, sampling strategies and the selection of villages were described elsewhere (Haque, 2012) [19]. Either oral or written consent was obtained from each participant. The study was approved by the Ethical Commission of Heidelberg University, Germany and, the research evaluation committee of the Department of Population Sciences, University of Dhaka, Bangladesh.

Both quantitative and qualitative instruments were used in the collection of data for the study. The validity and reliability of the instruments were insured by following a number of steps. First, a literature review was conducted to identify issues related to health coping strategies, health care providers, and sources of health care costs. Second, the survey questionnaires and interview guides for the focus group discussions (FGDs) and key informant interview (KII) were verified by experts in the field of health economics, public health and climate change. Third, the tools were pre-tested among 11 males and 9 females in the field and modified as needed before producing the final version.

Masters level students (2 males, 3 females) with fieldwork experiences administered the survey questionnaire. They were involved in the development of the data collection instruments which enabled them to understand the research concepts and questions. In addition, they were trained in building rapport, keeping confidentiality and maintaining social and cultural sensitivity at field level. First author (Haque, M. A.) was the team leader and present in the field full time to monitor the quality of the data collected. All surveys and interviews were administered in Bengoli. Translations of the themes to and from English were done by the first author.

The probability proportionate sampling (PPS) was used to maintain the proportionate number of households and respondents (male and female) to interview from each village. There were 1500 households in Dhuroil and 750 households in Sachibunia village (total 2250); from which, 460 households were selected randomly for interview. The response rate of the survey respondents was high (97.82%= 450) and no respondent discontinued the interview. The national male to female sex-ratio (51:49) was used in the calculations for selecting the number of male and female respondents from each village [35]. Either the eldest male or eldest female of the selected households were interviewed for the purpose of extensive information on their health coping strategies for climate induced diseases and sickness, the health care providers available to them and the resources used in covering the cost of health care.

The survey used a semi-structured interview schedule that included the background information of the respondent and their family members at the beginning of the interview. We assessed a detailed section including a total of 52 questions regarding various health coping strategies; self-reported measures or means the respondents used to avert climate-sensitive diseases and sickness. The solicited responses were categorical (“yes”, “no”, “don’t know”, “not applicable”). Quantitative data were analyzed using the Statistical Package for the Social Sciences (Version SPSS-12.0 and SPSS-17.0).

Qualitative data were collected through FGDs and KIIs. A total of 12 FGDs and 15 KIIs were completed by the research team using an interview guideline on three broad themes: health coping strategies, choice of treatments/care, choice of providers and health expenditure as included in the survey. Oral consent was taken from the participants before recording the interview and played back to them. Attending FGD and KII participants were “senior community members, farmers, non-governmental organization officials, village doctors, local political leaders and teachers of a socio-demographic background similar to that of the survey participants from the study areas” [19]. All FGDs and KIIs were transcribed and analyzed according to the broad themes: health coping strategies, choice of treatments/care, choice of providers and sources of the costs for health care.

## Results

### Socio-economic and demographic characteristics of the respondents

**Figure 1:**
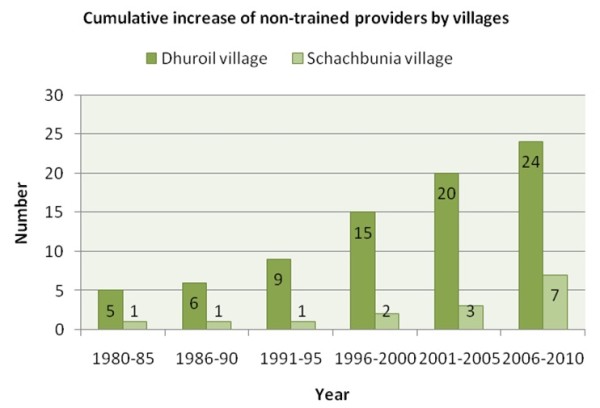
Cumulative increase of the number of the unqualified health providers by village.

All respondents came from agrarian-based rural areas of Bangladesh considered to be vulnerable to climate variability and changes. Fifty-three per cent of the survey respondents were male, and 47% were female. Twenty-seven per cent of the respondents had no formal education and 25% had only primary education. The mean age of males and females was 42 and 35 years, respectively (Table 1). The primary family occupation of all the respondents was farming. The median income was Bangladesh currency Taka (BDT) 7000 per month (US$1= BDT 74 in 2011) for a household of 4.15 persons. Per family average annual health expenditure for climate-sensitive diseases was BDT 9323. More details of the socio-demographic information of the respondents were presented in table one of Haque, et al. 2012 [19]. The villages were well connected with the Upazila (second level of administrative unit from bottom) and district headquarters. Each village was serviced by a UFHWC. Both constitute the only public medical facilities accessible to the villagers. Distance to the health center was within 1–2 kilometer from many households of the villages. Health care cost at the public health facilities are at free of cost. Public health facilities are run by government all over the country with qualified doctors, paramedics and nurses. In Dhuroil, the number of unqualified health providers rose from 5 in 1985 to 24 in 2010 while in Sachibunia the number increased from 1 to 7 over the same period (Figure 1). Unqualified providers are people who do not have a medical degree but who provide health or medical treatment to individuals. They have had a maximum of 3–6 months training in primary health care and locally known as ‘*palli*-*chikshok*’ (village doctor) in Bangladesh. In this study, we called them unqualified private providers. There was no private chamber of qualified providers. Nor were there any doctors in either village with a medical degree and training.

**Table 1:** Socio-demographic characteristics of the survey respondents (n=450)

**Category**	**By sex**		
	**Male**	**Female**	**Total**
N (%)	238 (53.0 )	212 (47.0)	450 (100.0)
Mean Age (Std.)	42 (13)	35 (10)	39.9
**Education**			
No Formal Education	62 (13.8)	59 (13.1)	121 (26.9)
Primary Education	60 (13.3)	52 (11.6)	112 (24.9)
Junior Secondary	38 (8.4)	56 (12.4)	94 (20.9)
Secondary School Certificate (SSC)	26 (5.8)	23 (5.1)	49 (10.9)
Higher Secondary (HSC)	23 (5.1)	13 (2.9)	36 (8.0)
Bachelor Level	20 (4.4)	5 (1.1)	25 (5.6)
Masters level	9 (2.0)	4 (0.9)	13 (2.9)
Total	238 (52.9)	212 (47.1)	450 (100.0)
**Occupation**	By Village		
	Dhuroil	Sachibunia	
Agricultural activities	116 (25.8)	24 (5.3)	140 (31.1)
Homemaker/Housewife	131 (29.1)	56 (12.4)	187 (41.6)
Services (Govt. NGO,)	22 (4.9)	26 (5.8)	48 (10.7)
Business (small and medium)	22 (4.9)	32 (7.1)	54 (12.0)
Others (village doctor, rickshaw puller, unemployed, fisherman)	6 (1.3)	15 (3.3)	21 (4.7)
Total	297 (66.0)	153 (34.0)	450 (100.0)
**Health Care Providers**	By village		
	Dhuroil	Sachibunia	Total
Union Family Health & Welfare Center	1	1	2
Satellite clinic	1	1	2
Village doctors (Pharmacies, Drug sellers)	24	7	31
Paramedics	2	1	3
**Household Information**			
Monthly median family income (BDT*)			7000
Mean family size (in persons)			4.15
Yearly average health expenditure/family			9323
Total health expenditure for all households (9323 X 2250)			2,09,77,200

* USD$1=BDT 74.00 in 2011; BDT= Bangladesh Currency (Taka).

#### Preventive health coping strategies

The respondents reported 22 types of different primary strategies to prevent themselves and their family members from the effect of climate change on their health (Table 2). Eleven of these health coping strategies dealt with heat, 6 from precipitation and 5 with cold events. In addition, the respondents approached neighbors, relatives, people who suffered from similar sickness, and NGO workers to discuss the climate-related health problems respondents faced and possible methods for preventing diseases/sickness (Table 2).

**Table 2:** Health coping strategies of the respondents for preventing sickness and diseases from extreme heat, cold and precipitation

**Preventive/Pre-sickness health coping strategies by the households**	**Yes**	
	n*	**%**
**What coping strategies did you adopt to avoid heat sensitive sickness during summer?**		
Finish all the tasks earlier in the morning	416	92.9
Do not get out when the temperature is too high	386	86.2
Do not get out during noon	395	88.2
Take extra rest at home	405	90.4
Do not go outside home unless urgent or necessary	406	90.6
Drink more sugar cane juice	372	83.0
Drink different homemade juices	431	96.2
Drink much water comparatively	444	99.1
Drink green coconut water	404	90.2
Try to keep sweating free and neat and clean	281	79.2
Take oral saline	205	57.7
**What coping strategies did you adopt to avoid precipitation sensitive sickness during rainy season?**		
Drink boiled water for drinking	45	10.0
Avoid using water from the river or pond	272	60.7
Use rain water	209	59.2
Try not to get wet in the rain	410	91.7
Don't let any water to stand beside the house	166	47.0
Use mosquito net to avoid the diseases	444	99.1
**What coping strategies did you adopt to avoid cold sensitive sickness during winter?**		
Drink much warm water or tea	305	68.1
Do not go out of the house until the sun comes out	210	46.9
Take shower with warm water	129	28.9
Use warm or heavy clothes to avoid cold	445	99.3
Use oil or body lotion to prevent skin diseases	435	97.1
**What additional coping strategies did you pursue other than seeking health care?**		
Discussed with neighbors	418	93.7
Got to know from someone who has suffered the same disease	312	72.2
Informed the relatives about it	357	84.2
Discussed with the NGO workers	22	5.4

n*= number.

(Q. What different coping strategies did you adopt to avoid climate sensitive sickness and diseases?).

#### Curative health coping strategies options and choices

**Figure 2:**
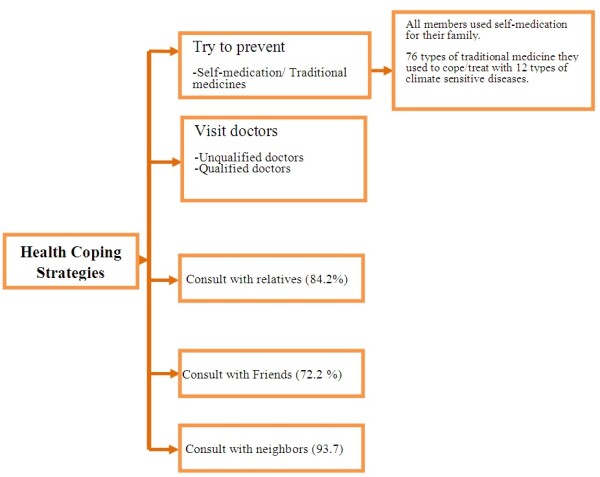
Health coping strategies used by the respondents to cope with climate-related health problems.

At the onset of disease and sickness 80.8% of the respondents used their self-knowledge of medication and previous healing experiences to treat themselves and their family members; 99.3% sought treatment available in the rural area (Table 3). In response to the question “*what were the choices among the health coping strategies in case of climate sensitive diseases or sickness for your family members*,” 54.4% of the participants reported that self-knowledge or home-remedies and previous healing experiences were the first strategies they used in treating climate related health problems. Approximately 43.3% of the respondents sought any treatment (which included qualified, unqualified providers) as their first choice. Respondents used multiple means to cope with climate sensitive health problems (Figure 2).

**Table 3:** Health coping strategies by the survey participants (n=450)

**Q: what did you do in case of climate sensitive diseases or sickness among your family members?**			**Q: what were the choices of the strategies for health coping?**					
			**1st choice**		**2nd choice**		**3rd choice**	
**Strategies for coping with health problems**	**Yes**		**Yes**		**Yes**		**Yes**	
	n*	**%**	**n**	**%**	**n**	**%**	**n**	**%**
Applied personal experiences & knowledge	363	80.8	245	54.4	119	26.4	0	0.0
Sought treatment (qualified/unqualified treatment)	446	99.3	195	43.3	246	54.7	3	0.7
Wanted to but could not afford	9	2.0	7	1.6	2	0.4	3	.7
Did nothing	3	0.7	3	0.7	1	0.2	23	5.1
Total			450	100.0				
*n=number								

(Q: what did you do in case of climate sensitive diseases or sickness among your family members? and.

Q: what were the choices of the strategies for health coping?).

#### Health care providers options for health coping

There were 12 types of health providers available in the study areas (Table 4). Most of them were unqualified health care providers. They included village doctors, drug stores, folk medicine, family welfare assistants/visitors, homeopath and spiritual healers. The percentage of visiting the village doctors was 92.7, drug stores 75.9 and self-medication was 67.3. Unqualified providers were people from within the villages, known to them, they were open for whole day (morning, afternoon, evening), and people could negotiate their payment and buy medicine in credit. The use of qualified providers (health services at Upazila Health Complex and UFHWC) was low compared to unqualified providers. Only 30.3% respondents reported that they had visited the Upazila Health Complex (UHC) for treatments. The UHC is the second level of the health structure of government. It has specialized health services for inpatients with outpatient/outdoor services. A KII informed that “its waste of time visiting Union or Upazila health facilities as there is no medicine supply, no doctor, and no treatment when we visit. We are not sure that we will get treatment what we need”.

**Table 4:** Types of health care providers visited (n=450)

**Health Care Providers**					
**Unqualified providers (UQP)**	**Yes**		**Qualified Providers (QP)**	**Yes**	
	n*	**%**		**n**	**%**
Village doctor	416	92.7	**Public**		
Drug stores	341	75.9	Upazila Health Complex	136	30.3
Self-medication	302	67.3	Union Health/Satellite Clinic	100	22.3
FWA/FWV/Nurses	161	35.9	**Private**		
Medical assistant	39	8.7	Private clinic	160	35.5
Homeopath	158	35.2	Paramedics	266	59.1
Spiritual treatment	94	20.9			
Folk medicine	142	31.6			
*n=number					

Q: Where did you go for the treatments for your family members in case of climate sensitive diseases and sickness?

#### Choices of health provider for coping

**Figure 3:**
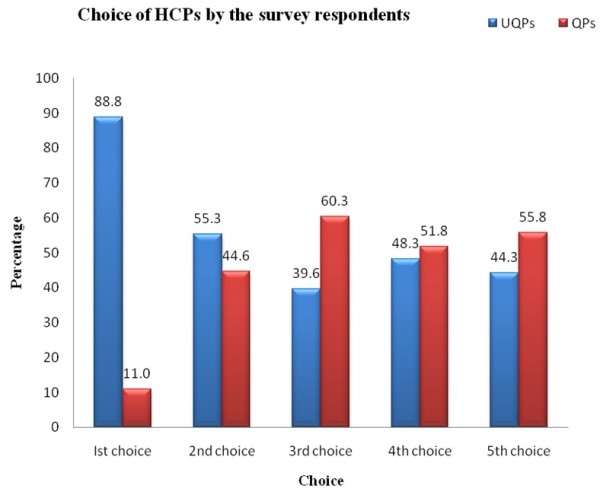
Choice of the health care providers (HCPs) by the survey participants.

Eighty-eight per cent of the respondents choose unqualified health providers as their first choice and 54.2% as their second choice (Table 5). The use of qualified providers increased from 11% as their first choice to 44.6% as their second choice. Qualified providers were primarily their fourth and fifth choices. A noticeable finding was that public health facilities were never among respondents’ first choices for treatment. The percentage of using qualified providers increased to the second and third-choice categories, while the percentage of using unqualified providers decreased to the same choices. About 60% of the respondents had selected qualified providers as their third choice. Gradually, from second choice onward, respondents preferred qualified providers as their health coping strategy (Figure 3) for their climate sensitive diseases or sickness.

**Table 5:** Range of health care options chosen by interview respondents (n=450)

**Different health care providers**	**Choices of providers**									
	**1**^**st**^		**2**^**nd**^		**3rd**		**4th**		**5th**	
	**Yes**		**Yes**		**Yes**		**Yes**		**Yes**	
	n*	**%**	**n**	**%**	**n**	**%**	**n**	**%**	**n**	**%**
**Unqualified health providers**										
Village doctor	294	65.3	118	26.2	47	11.3	46	18.2	17	19.8
Drug store	44	9.8	45	10.0	33	8.0	10	4.0	0	0
Folk medicine	5	1.1	36	8.0	33	8.0	6	2.4	1	1.2
Self-Medication	51	11.3	27	6.1	11	2.7	5	2.0	0	0
Family Welfare Assistant/Visitors	2	0.4	9	2	6	1.4	5	2	0	0
Homeopath	3	0.7	8	1.8	29	7.0	39	15.4	12	14.0
Spiritual treatment	1	0.2	1	.2	5	1.2	11	4.3	8	9.3
***Subtotal of UHP***	**400**	**88**.**8**	**244**	**54**.**2**	**164**	**39**.**6**	**122**	**48**.**3**	**38**	**44**.**3**
**Qualified health providers**										
Upazila health complex/MBBS	3	0.6	103	23.4	174	42	78	30.8	29	33.7
Union health/Satellite Clinic	0	0	18	4.1	18	4.3	7	2.8	2	2.3
Private Clinic	2	.4	6	1.4	17	4.1	28	11.1	15	17.5
Paramedics	45	10.0	69	15.7	41	9.9	18	7.1	2	2.3
**Subtotal of QHP**	**50**	**11**.**0**	**196**	**44**.**6**	**250**	**60**.**3**	**131**	**51**.**8**	**48**	**55**.**8**

n=number.

Q: What was your choice of health providers for your family members in case of climate sensitive diseases and sickness?

#### Sources of money for health coping

All the respondents reported that they had to manage health care expenditure to cope with climate related diseases personally. Only 46% of the money which family members spent was from savings. The rest, 56%, had to be acquired from different informal sources outside the family (Table 6). A total of 24 types of different informal sources were reported. The sources included loans taken from *mahajans* (person who gives loan among villagers informally with high interest/money lender), businessmen, NGOs, banks, neighbors, relatives, or drug stores, as well as the money received from the sale of domestic animals, agricultural crops, ornaments, or from religious funds (known as *zakat*, *fetra*). Some families were given donations from neighbors and relatives. The use of “social capital” [37-41], i.e. borrowing money from neighbors, relatives and drug stores were the most common source of money for health expenditure of the respondents. Community-fund pooling, common funding, and health insurance did not factor as sources for the respondents’ climate related health care expenditure. Neither public nor private health insurance systems were available to the participants in the study areas.

**Table 6:** Methods used by interview respondents to obtain money for health care services to cope with climate-related health problems

**Sources of money for health expenditure**	**n**	**% of Respondents**	**Total amount spent**	**Amount spent from different sources (%)**
Had my own money	450	100.0	1,949,080	46.46
Took loan from *Mahajans*	8	1.8	31,000	0.74
Took loan from businessman	4	0.9	14,500	0.35
Took loan from NGO	34	7.6	171,200	4.08
Took loan from banks	6	1.3	44,000	1.05
Spent NGO loans taken for other purposes	5	1.1	12,700	0.30
Lent from neighbors	185	41.1	310,330	7.40
Lent from relatives	168	37.3	407,130	9.70
Lent from drug stores	121	26.9	78,100	1.86
Sold cattle	63	14.0	372,900	8.89
Sold crops	112	24.9	310,850	7.41
Sold land	12	2.7	103,700	2.47
Sold trees	12	2.7	36,400	0.87
Sold ornaments	9	2.0	34,700	0.83
Sold fish in advance	4	0.9	11,000	0.26
Sold fruits of own trees	4	0.9	4,000	0.10
Sold poultry	16	3.6	10,950	0.26
Took *Jakat*/*Fetra* (*religious fund*/*support*)	4	0.9	1,600	0.04
Sought financial help	15	3.3	68,800	1.64
Begged	2	0.4	2,000	0.05
Took relief from Government and public	1	0.2	1,500	0.04
Mortgaged Land	22	4.9	208,000	4.96
Leased land	2	0.4	8,000	0.19
Leased Ornaments	2	0.4	3,000	0.07
Insurance/community funding	0	0.0	0	0.0
Total			4,195,440	100.00

Q: What were the sources of health expenditure (yearly) for your family members for the treatment of climate sensitive diseases and sickness?

## Discussion

This study’s findings provide important insights into what people of a resource-poor setting do when they are affected by climate change exacerbated sicknesses and illnesses. The respondents were from the rural areas which are vulnerable to climate change [42]. Most of the respondents had agricultural occupations and, a low level of formal education and family income. Almost all those surveyed, including focus group discussions and key informants, reported that diseases and sickness had increased due to climate change (changes in heat, cold and precipitation) [19]. The study also informs our understanding about the health coping strategies of the respondents, i.e. preventive health coping options and available and preferred types of health care providers. It also enables us to discern the choices of health care seeking; per family climate related health expenditures (HE) [43]; and the different sources of HEs whose health was negatively affected by climate change [19] in a rural setting.

Almost all respondents sought an array of preventive measures to protect their health from the effect of extreme climate change. They choose at least one type of health care service for coping with climate sensitive health problems. As curative health coping strategies, most of the respondents sought treatment from the available unqualified providers in the villages. Only very few could not seek any kind of treatment due to financial inability. The Bangladesh government has decentralized health care facilities up to Union and village levels to introduce quality and trained health care facility officials in villages. It has also tried to ensure accessibility and availability of health care services to people in rural and urban communities [44]. This, however, is not reflected in the findings of the study [45]. Visiting public health facilities were not factored among the first, second or third choice strategies of the villagers in coping with their climate induced health problems. Unqualified providers played a dominant role among the respondents.

The estimated annual health expenditure of all the households (2250) in the two villages in treating the diseases induced by climate change was about BDT 20,977,200 (annual HE × all households), which was spent primarily on unqualified providers. Although respondents were from low income and climate vulnerable groups, they paid high prices for low quality health services from unqualified providers with all the money coming from out-of-pocket (OOP) payment. Strong initiatives from government of the country, international development partners and NGOs are needed to motivate people for the effective use of money they spent individually and to introduce community-based health coping strategies. If community pooling, prepaid health care or insurance system could be introduced at the community level to pool this amount from the households, quality health care services could be provided at the rural level. Such pooling also can help the villagers cope with the additional cost of climate change exuberated health impacts and help achieve funding for universal coverage [46]. The lack of socialized fund-pooling mechanisms exacerbates the situation as high costs might exclude poor people from access to effective health care [47]. Latko B et al. 2011 stated that “taking money from poor people when they are sick is not a good idea” [48], this inequality needs to be addressed by health system reform [49]. Informing the respondents about their high spending could motivate them to develop community fund pooling, health insurance or pre-paid health care with special attention to climate-sensitive health problems. It may also inspire them to enroll for health insurance, thus ensuring adequate and quality health care among rural climate vulnerable people.

Limited income and high prices influence the access to health care of people [50,51]. There was no health insurance or community funding [48] in the community and no NGO initiative to support the high OOP payment and health coping costs of the respondents. There was a great uncertainty in managing climate related health coping costs among the respondents. As a consequence, direct OOP payment spending and the use of “social capital” [52-55] represented the few options available to purchase health care for coping with climate change exuberated diseases and sickness. Findings show that OOP payment among the respondents is even higher than what was calculated in the national health account (NHA) [46,56]. Collection of such research findings can provide necessary information for health economists to recalculate the direct health expenditures of the people vulnerable to climate change in Bangladesh and NHA [56]. The higher the magnitude of climate change induced diseases, the more socio-economic losses of the households for health coping will accrue. The increase of climate sensitive diseases and almost exclusive out-of-pocket spending for coping with health problems will make the villagers more vulnerable [49].

The study included data from two rural villages among many that actually exist in Bangladesh; as such, the results may not be generalizable to rural communities across the country. The generalization of these health coping strategies to other areas of Bangladesh may require further research. Additionally, there might be recall bias as well as a social desirability bias, as we also had to depend on the subjective judgments of the respondents’ experiences on health coping.

## Conclusions

People of the 2 rural communities in Bangladesh included in this study are concerned about climate induced diseases and sickness and sought preventive as well as curative measures to cope with health problems. Every respondent used traditional knowledge and known health care practices to cope with climate sensitive health problems. Seeking health care from unqualified private health care providers is the most commonly used most available health coping strategy in treating sicknesses and illnesses brought on by climate change. Public health care facilities at the community level are not used by the respondents to cope with the same health problems. Per family spending to cope with such health problems is very high and health care is solely based on out of pocket payment. Most of them had to depend on their available family assets as well as their “social capital” to cope with climate related health problems. There is no fund pooling, community funding or health insurance program in the study areas to support the overall health coping of the climate vulnerable people. Initiatives and strong advocacies are needed from the government, NGOs and development partners to improve the health coping options for the people vulnerable to climate change in the rural areas. They also need to set measures, to reduce OOP payments and high health care costs and to improve the health services at public and private levels. Such measures are necessary for helping the people vulnerable to climate change in resource poor settings to cope with additional climate induced health problems. Coping with climate related health problems at the village level is mostly an individual’s responsibility that is, dependent on unqualified treatments at high prices. There is neither community based mechanism to cope with climate induced health problems nor any additional program or support from the government. The collection of such information on climate related health coping can benefit the government, NGOs and development partners in formulating strategies for effectively coping with the climate induced diseases and sickness.

## Abbreviations

FGD: Focus Group Discussion; KII: Key Informant Interview; IPCC: Inter-governmental Panel on Climate Change; WHO: World Health Organization; UFHWC: Union Family and Health Welfare Center; UHC: Upazila Health Complex; CCC: Climate Change Cell; US$: United State Dollar; UNFPA: United Nation’s Population Fund; EMMA: Erasmus Mundus Mobility in Asia; OOP: Out of Pocket; NHA: National Health Account; NGO: Non-Government Organization; UQHP: Unqualified health provider; QHP: Qualified health provider; HCP: Health care provider.

## Competing interests

The authors declare that they have no competing interests.

## Authors’ contributions

MAH designed the study, developed the questionnaire, supervised the data collection, analyzed the data and wrote the paper. BA, contributed to interpret the results. SSY and VL contributed to the drafting and writing of the manuscript. AAM contributed to the study design. RS contributed to the development of the overall study concept, design of the study and drafting of the paper. All authors read and approved the final manuscript.

## Pre-publication history

The pre-publication history for this paper can be accessed here:

http://www.biomedcentral.com/1471-2458/13/565/prepub

## References

[B1] IPCC Climate change 2001: the scientifc basis. Contribution of working group I to the Third Assessment Report of the Intergovernmental Panel on Climate Change 2001 Cambridge: Cambridge University Press

[B2] IPCC Intergovernmental Panel on Climate Change. Climate change 2007: the physical science basis. 2007 2007 http://www.ipcc.ch/ipccreports/ar4-wg1.htm (accessed Sept 30, 2009)

[B3] MoEF National Adaption Program of Action (NAPA) 2005 Dhaka: Ministry of Environment and Forest 1 63

[B4] UNDP Atiq Rahman MRU, Mozaharul A, Sarder Shafiqul A, Golam R, Ariam R Background Paper on Risks, Vulnerability and Adaptation in Bangladesh Human Development Report 2007 2007 Dhaka

[B5] UNDP Study on Perception of Illness and Health Seeking Behavior among Five Ethnic Groups 2010 Dhaka: UNDP

[B6] Rahman A Climate change and its impact on health in Bangladesh Regional Health Forum - Volume 12, Number 1, 2008 2008 2 1 16 26

[B7] Shahid S Probable Impacts of Climate Change on Public Health in Bangladesh Asia Pac J Public Health 2009 23 3 1 10 10.1177/1010539509335499 19443872

[B8] Hashizume M Armstrong B Hajat S Wagatsuma Y Faruque ASG Hayashi T Sack DA Association between climate variability and hospital visits for non-cholera diarrhoea in Bangladesh: effects and vulnerable groups Int J Epidemiol 2007 36 5 148 10.1093/ije/dym148 17664224

[B9] Preet R Nilsson M Schumann B Evengård B The gender perspective in climate change and global health Global Health Action 2010 3 10.3402/gha.v3i0.5720 PMC3001868 21160554

[B10] Confalonieri U Menne B Akhtar R Ebi KL Hauengue M Kovats RS Revich B Woodward A Parry ML, Canziani OF, Palutikof JP, Linden PJ, Hanson CE Impacts, Adaptation and Vulnerability. Contribution of Working Group II to the Fourth Assessment Report of the Intergovernmental Panel on Climate Change Human health. ClimateChange 2007 2007 Cambridge, UK: Cambridge University Press 391 431

[B11] Hales S Weinstein P Woodward A Public health impacts of global climate change Rev Environ Health 1997 12 3 191 199 9406290 10.1515/reveh.1997.12.3.191

[B12] Ebi KL Semenza JC Community-Based Adaptation to the Health Impacts of Climate Change Am J Prev Med 2008 35 5 501 507 10.1016/j.amepre.2008.08.018 18929976

[B13] Costello A Maslin M Montgomery H Johnson AM Ekins P Global health and climate change: moving from denial and catastrophic fatalism to positive action Philos Transact A Math Phys Eng Sci 2011 369 1942 1866 1882 10.1098/rsta.2011.0007 21464077

[B14] Kovats S Haines A The potential health impacts of climate change: an overview Medicine and war 1995 11 4 168 178 10.1080/07488009508409236 8559115

[B15] Bush KF Luber G Kotha SR Dhaliwal RS Kapil V Pascual M Brown DG Frumkin H Dhiman RC Hess J Impacts of climate change on public health in India: future research directions Environ Health Perspect 2011 119 6 765 770 10.1289/ehp.1003000 21273162 PMC3114809

[B16] Health impacts of climate change Chemical & Engineering News 2008 86 15 34 3423776841

[B17] WHO Protecting Health from Climate Change - World Health Day 2008 2008 Swetzerland: World Health Organization

[B18] WHO Gender Climate Change and Health 2009 Geneva: World Health Organization

[B19] Haque MA Yamamoto SS Malik AA Sauerborn R Households' Perception of Climate Change and Human Health Risks: A community perspective Environ Health 2012 11 1 1 10.1186/1476-069X-11-1 22236490 PMC3311088

[B20] IPCC Climate change: impacts, adaptation, and vulnerability. Contribution of Working Group II to the third assessment report of the Intergovernmental Panel on Climate Change 2001 New York: Cambridge University Press 1032

[B21] Ebi K Woodruff R von Hildebrand A Corvalan C **Climate Change-related Health Impacts in the Hindu Kushira Himalayas** EcoHealth 2007 4 3 264 270 10.1007/s10393-007-0119-z

[B22] Khan AE Ireson A Kovats S Mojumder SK Khusru A Rahman A Vineis P Drinking Water Salinity and Maternal Health in Coastal Bangladesh: Implications of Climate Change Environ Health Perspect 2011 119 9 1328 1332 10.1289/ehp.1002804 PMC3230389 21486720

[B23] World Health O Health and Environmental Linkage Initiative. Climate Change: deaths from climate change 2011 1 2 (http://www.who.int/heli/en/)

[B24] McMichael AJ Butler CD Health Promotion Challenges: Emerging health issues: the widening challenge for population health promotion Health Promotion International 2007 21 S1 15 24 17307953 10.1093/heapro/dal047

[B25] Rahman A Bangladesh's role on Climate Negotiation 2011 Dhaka: The Daily Star

[B26] Forests ME National Adaption Program of Action (NAPA) 2005 Dhaka: Ministry of Environment and Forests. Government of the People's Republic of Bangladesh 1 63

[B27] IPCC Climate change 2001: impacts, adaptation, and vulnerability. Contribution of Working Group II to the third assessment report of the Intergovernmental Panel on Climate Change (IPCC) 2001 New York: Cambridge University Press 1032

[B28] Ministry of H, Family W Global Climate Change: Health Impacts on Bangladesh. Pocket Book 2009 2009 Dhaka: Ministry of Health and Family Welfare. Government of the People's Republic of Bangladesh 1 38

[B29] GermanWatch Global Climate Risk Index 2009. Weather-Related Loss Events and their Impacts on Countries in 2007 and in A Long-Term Comparison 2007 Germany: GermanWatch

[B30] CCC Climate Change in Bangladesh 2007 Government of the People's Republic of Bangladesh: Dhaka: Ministry of Environment and Forest 1 24

[B31] CCC Climate Change and Health Impacts in Bangladesh Climate Change Cell (CCC), Ministry of Envirnment and Forest 2009 Dhaka, Bangladesh: Government of the People's Republic of Bangladesh 1 82

[B32] Morse MJ Niehaus L Mixed Method Design: Principles and Procedures (Developing Qualitative Inquiry) 2009 Walnut Creek, California: Left Coast Press

[B33] Creswell WJ Research Design: Qualitative, Quantitative, and Mixed Methods Approaches, vol. Third 2009 New Delhi: Sage Publications, Inc

[B34] Creswell WJ Qualitative Inquiry & Research Design: Choosing Among Five Approaches, Volume 2nd 2007 New Delhi: Sage Publications, Inc.

[B35] BBS Population Census Report 2011 2011 Dhaka: Ministry of Planning. Government of the People's Republic of Bangladesh

[B36] National Institute of Population R, Training, Mitra, Associates, and Macro Bangladesh Demographic and Health Survey 2007 2009 Dhaka: NIPORT, Bangladesh and Calverton, Maryland, USA

[B37] Bank W What is Social Capital Online (Accessed on 27 Feb 2012): The World Bank

[B38] Poortinga W Community resilience and health: The role of bonding, bridging, and linking aspects of social capital Health Place 2012 18 2 286 295 10.1016/j.healthplace.2011.09.017 22037322

[B39] Protecting Health from Climate change. Global Research Priorities 2009 WHO

[B40] Hunter BD Neiger B West J The importance of addressing social determinants of health at the local level: the case for social capital Health & social care in the community 2011 19 5 522 530 10.1111/j.1365-2524.2011.00999.x 21595772

[B41] Barnett R Coping with the costs of primary care? Household and locational variations in the survival strategies of the urban poor Health & Place 2001 7 2 141 157 10.1016/S1353-8292(01)00013-2 11470227

[B42] National Adaption Program of Action 2005 Government of the People's Republic of Bangladesh: Ministry of Environment and Forests 1 63

[B43] Turner GM California and universal health coverage MedGenMed : Medscape general medicine 2007 9 1 36 17435642 PMC1924988

[B44] Barbagli M Santoro M Le basi morali dello sviluppo: capitale sociale, criminalità e sicurezza in Sardegna 2004 1 Cagliari: AM&D

[B45] Frenk J Gomez-Dantes O Knaul FM The democratization of health in Mexico: financial innovations for universal coverage Bull World Health Organ 2009 87 7 542 548 10.2471/BLT.08.053199 19649369 PMC2704036

[B46] Garrett L Chowdhury AMR Pablos-Méndez A All for universal health coverage The Lancet 2009 374 9697 1294 1299 10.1016/S0140-6736(09)61503-8 19698983

[B47] Mathauer I Carrin G The role of institutional design and organizational practice for health financing performance and universal coverage Health Policy 2011 99 3 183 192 10.1016/j.healthpol.2010.09.013 20965603

[B48] Latko B Temporao JG Frenk J Evans TG Chen LC Pablos-Mendez A Lagomarsino G de Ferranti D The growing movement for universal health coverage Lancet 2011 377 9784 2161 2163 10.1016/S0140-6736(10)62006-5 21084114

[B49] Hu S Universal coverage and health financing from China's perspective Bull World Health Organ 2008 86 11 819 10.2471/BLT.08.060046 19030679 PMC2649565

[B50] Semitiel García M Social capital, networks and economic development : an analysis of regional productive systems 2006 Cheltenham, UK: Northampton, MA: Edward Elgar

[B51] Durlauf SN Fafchamps M Social capital NBER working paper series working paper 10485 2004 Cambridge, MA: National Bureau of Economic Research

[B52] Card D Dobkin C Maestas N The impact of nearly universal insurance coverage on health care utilization: evidence from medicare The American economic review 2008 98 5 2242 2258 10.1257/aer.98.5.2242 19079738 PMC2600774

[B53] Kawachi I Subramanian SV Kim D Social capital and health 2008 New York; London: Springer

[B54] Brennan VM Natural disasters and public health: Hurricanes Katrina, Rita, and Wilma 2009 Baltimore: Johns Hopkins University Press

[B55] Watkins D Cousins J Public health and community nursing: frameworks for practice 2010 3 Edinburgh, New York: Elsevier Bailliere Tindal

[B56] MoHaF Bangladesh National Health Accounts 1997–2007 2007 Dhaka: Ministry of Health and Family Wealfare (MoHFW) 1 107

